# Abnormal fetal movements, micrognathia and pulmonary hypoplasia: a case report. Abnormal fetal movements

**DOI:** 10.1186/1471-2393-10-46

**Published:** 2010-08-17

**Authors:** Seiichi Morokuma, Ai Anami, Kiyomi Tsukimori, Kotaro Fukushima, Norio Wake

**Affiliations:** 1 Institutional Affiliations: Department of Obstetrics and Gynecology, Graduate School of Medical Sciences, Kyushu University, Japan

## Abstract

**Background:**

Micrognathia is a facial malformation characterized by mandibular hypoplasia and a small, receding chin that fails to maintain the tongue in a forward position. We previously reported a system of prenatal screening that we developed to identify fetuses with compromised central nervous system function by observing fetal behavior. In this paper we report the case of a preterm infant with micrognathia and pulmonary hypoplasia who presented abnormal fetal movements.

**Case presentation:**

A 27-year-old Japanese primigravida at 33 weeks of gestation was referred to our hospital. Ultrasonographic examination revealed clinical polyhydramnios. Micrognathia was evident on midsagittal and 3 D scan. The lung area was less than the mean -2.0 standard deviations for the gestational age. The infant had mandibular hypoplasia and glossoptosis. After emergency cesarean delivery for non-reasuring fetal status, required immediate tracheostomy and cardiopulmonary resuscitation with mechanical ventilatory support. However, the infant's cardiopulmonary condition did not improve and she died 21 hours after birth.

**Conclusions:**

The findings of our ultrasound exam are suggestive of brain dysfunction. The observation of fetal behavior appears to be effective for the prediction of prognosis of cases with micrognathia.

## Background

Micrognathia is a facial malformation characterized by mandibular hypoplasia and a small, receding chin that fails to maintain the tongue in a forward position. Conditions associated with micrognathia include various abnormalities, and the prognosis of fetal micrognathia is poor, even in chromosomally normal fetuses [1,2]. When micrognathia is isolated, it is considered a component of Pierre-Robin syndrome (PRS) [1]. The underlying etiology of PRS has not yet been well established. Mandible growth results from oral motility, which begins during early fetal life [3].

Previously, we reported a system of prenatal screening that we developed to identify fetuses with compromised central nervous system function by observing fetal behavior [4]. We report the case of a preterm infant with micrognathia and pulmonary hypoplasia who presented abnormal fetal movements.

## Case presentation

**Figure 1:**
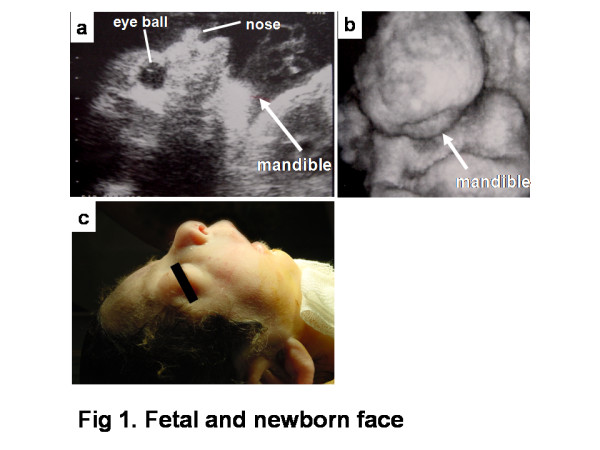
**Fetal and newborn face**. B-mode ultrasound scan of the fetal face at 33 weeks of gestation showing micrognathia (arrows: mandible) (a). Three-dimensional ultrasound scan of the fetal face at 33 weeks of gestation showing micrognathia (arrows: mandible) (b). Newborn face showing micrognathia (c).

A 27-year-old Japanese primigravida at 33 weeks of gestation was referred to our hospital with polyhydramnios and threatened preterm labor. Ultrasonographic examination revealed clinical polyhydramnios (amniotic fluid index: 28 cm). Micrognathia was evident on midsagittal and 3 D scan (fig 1-a, b). The lung area of 8.9 cm2 in the four-chamber view was less than the mean -2.0 standard deviations for the gestational age (normal; mean ± 2SD, 20.1 ± 7.6). The biparietal diameter was 82 mm, femur length 51 mm, and the estimated fetal weight was 1,500 g, suggesting fetal growth restriction. Pulsed Doppler sonography showed normal middle cerebral artery and umbilical artery pulsatility indices. Amniocentesis was performed for a chromosome study, with a result of a 46, XX karyotype. We observed fetal movements for 90 minutes at 34 weeks 3 days of gestation. Movement in all four extremities was observed; however, no breathing or mouthing movements were detected, and the fetus had sporadic eye movements. At 34 weeks 5 days of gestation, a cesarean section was performed for non-reassuring fetal status. The female infant had a birth weight of 1,675 g, with an umbilical artery pH of 7.385. Apgar scores were 5 at one minute and 7 at five minutes. The infant had mandibular hypoplasia and glossoptosis and was diagnosed with PRS. Severe respiratory compromise required immediate tracheostomy and cardiopulmonary resuscitation with mechanical ventilatory support. The infant's cardiopulmonary condition did not improve, and she died 21 hours after birth.

The infant did not have microcephaly, dysmorphic features, or hand/foot anomalies.

At autopsy, the lungs contained little air, and the lung to body weight ratio was 0.01. Histologically, the epithelium of the pulmonary alveoli was thick and dysplastic as well as reduced in number. These findings correlated with a lung maturity of 17-24 weeks of gestation. In addition, no abnormalities of the internal organs were observed.

## Conclusions

Conditions associated with micrognathia include chromosomal abnormalities, neuromuscular abnormalities, single-gene disorders, and other syndromes. The prognosis of fetal micrognathia is poor, even in chromosomally normal fetuses [2]. In this report, we have described a case of micrognathia associated with pulmonary hypoplasia. No reports of micrognathia associated with pulmonary hypoplasia in the absence of chromosomal abnormalities or Pena-Shokeir syndrome have been published. The present case had no chromosomal abnormalities and showed movement of the extremities, and it is not likely to have had a single-gene disorder or other syndrome, as no microcephaly, dysmorphic features, or hand/foot anomalies were observed.

This case showed abnormal behavioral patterns, including sporadic eye movements, which were documented on a prenatal ultrasound exam. Normal alternations of eye movement and non-eye movement periods as well as breathing and mouthing movements were not evident. Movements of the extremities were observed. In animals, the neural center that generates the alternation rhythm of the eye movement and non-eye movement periods lies within the pons and/or medulla oblongata [4]. The absence of fetal breathing movements suggests a lesion involving the medulla oblongata, the breathing center. Thus, we suspected brainstem dysfunction prenatally.

Abadie et al. proposed that dysfunction of the brainstem region controlling the rhythmic reflex of sucking and swallowing, cardiorespiratory, pharyngeal, and laryngeal functions may contribute to the severe feeding and respiratory disorders seen in infants with PRS. These functional anomalies involve several organs controlled by common neuronal networks located in the brainstem. Micrognathia results from a lack of mandibular movements and respiratory movements that are required for lung development. These investigators have suggested a prenatal and neonatal brainstem dysfunction as a "neuroembryological hypothesis" to explain the onset of some cases of PRS [5,6]. The structural and functional abnormalities observed during our ultrasound examination are consistent with this idea.

In our case, a postmortem brain examination was not performed. However, the findings of our ultrasound exam are suggestive of brain dysfunction. The observation of fetal behavior appears to be effective for prediction of prognosis of cases with micrognathia.

## Consent

Written informed consent was obtained for publication of this case report and accompanying images. A copy of the written consent is available for review by the Editor-in-Chief of this journal.

## Competing interests

The authors declare that they have no competing interests.

## Authors' contributions

SM, AA and KT examined the findings of this case and drafted the manuscript. KF and NW participated in the design of the study and coordination. All authors read and approved the final manuscript.

## Pre-publication history

The pre-publication history for this paper can be accessed here:

http://www.biomedcentral.com/1471-2393/10/46/prepub
